# Local Initiatives to Fund Services for Elders: Increased Community Recognition of the Importance of Social Care

**DOI:** 10.1093/geroni/igaa057.339

**Published:** 2020-12-16

**Authors:** Athena Koumoutzis, Sara Stemen, Renusha Maharjan, Jennifer Heston-Mullins, Pamela Mayberry, Robert Applebaum

**Affiliations:** Miami University, Oxford, Ohio, United States

## Abstract

Despite the growing number of older adults in the U.S., federal and state funding for non-medical supportive services remains limited. Recent work reports that states with a more generous supply of supportive services, including home delivered meals and personal care, have fewer low care residents in nursing homes. To boost this supply, some local communities across the nation are exploring alternative funding sources. Our review found 400 local communities across 15 states using voter-approved local revenue streams to fund aging services, such as property tax levies and payroll and sales taxes, and that this strategy has been politically popular. In this paper we provide results from the first national survey of these local communities. Study results found considerable variation by state in number and scope of local initiatives, with Ohio and Michigan each reporting about 70 communities with local property tax levies, while California and Washington had only one community each using this approach. Local programs ranged in size from generating less than $25,000 in annual revenue to more than $35 million. The organizational structure for these programs, and the administrative approaches, such as the use of care managers, varied by state and community. Programs provided an array of services, but typically included traditional social care services such as home delivered meals, homemaker/personal care, transportation, and home emergency response systems. Criteria for program participation also varied, but most were targeted to serve older adults with disability who did not meet Medicaid financial or functional eligibility criteria.

